# Involvement of Nitric Oxide in TRPV4-Induced Relaxations of Mouse and Human Pulmonary Arteries

**DOI:** 10.3390/biology15030292

**Published:** 2026-02-06

**Authors:** Vytis Bajoriūnas, Agilė Tunaitytė, Augusta Volkevičiūtė, Silvijus Abramavičius, Ieva Bajoriūnienė, Edgaras Stankevičius, Ulf Simonsen

**Affiliations:** 1 Preclinical Research Laboratory for Medicinal Products, Institute of Cardiology, Lithuanian University of Health Sciences, Sukileliu Ave. 13, LT-50166 Kaunas, Lithuania; vytis.bajoriunas@lsmu.lt (V.B.); augusta.volkeviciute@lsmu.lt (A.V.); silvijus.abramavicius@lsmu.lt (S.A.); edgaras.stankevicius@lsmu.lt (E.S.); 2 Heart Centre, Faculty of Medicine, Medical Academy, Lithuanian University of Health Sciences, LT-50161 Kaunas, Lithuania; 3 Institute of Physiology and Pharmacology, Faculty of Medicine, Medical Academy, Lithuanian University of Health Sciences, LT-44307 Kaunas, Lithuania; 4 Department of Immunology and Allergology, Faculty of Medicine, Medical Academy, Lithuanian University of Health Sciences, LT-50161 Kaunas, Lithuania; ieva.bajoriuniene@lsmu.lt; 5 Department of Biomedicine, Pulmonary and Cardiovascular Pharmacology, Aarhus University, Høgh-Guldbergsgade 10, 8000 Aarhus C, Denmark; us@biomed.au.dk

**Keywords:** nitric oxide, NO synthesis, pulmonary arteries

## Abstract

The transient receptor potential vanilloid 4 channel (TRPV4) is thought to play a pivotal role in pulmonary arterial circulation. The present study hypothesizes that TRPV4 activation increases nitric oxide (NO) release and activates calcium-activated potassium of intermediate conductance (KCa3.1) in pulmonary arteries. Pulmonary arteries were isolated from wild-type mice (wt) and mice deficient in KCa3.1 channels (*Kcnn4*^−/−^) and mounted for simultaneous NO concentration and relaxation measurements. Human small pulmonary arteries were isolated and mounted in microvascular myographs for isometric tension recordings. Acetylcholine-induced increases in NO and relaxation of pulmonary arteries were slightly decreased in pulmonary arteries from *Kcnn4*^−/−^ versus wt mice. An activator of TRPV4 channels, GSK1016790A, increased NO and relaxation to the same degree in pulmonary arteries from wt and *Kcnn4*^−/−^ mice. A blocker of TRPV4 channels, HC06704, inhibited increases in NO concentration with no effect on acetylcholine (ACh) relaxation in pulmonary arteries from wt mice, but blocked increases in NO concentration and relaxation in pulmonary arteries from *Kcnn4*^−/−^ mice and responses to GSK1016790A in pulmonary arteries from wt and *Kcnn4*^−/−^ mice. Concentration-dependent relaxations induced by an inhibitor of sarcoplasmic Ca-ATPase, cyclopiazonic acid, were blocked in the presence of an inhibitor of NO synthase and a blocker of KCa3.1 channels, TRAM-34, in pulmonary arteries from wt mice, but were unaltered in the presence of TRAM-34 in arteries from *Kcnn4*^−/−^ mice, or the presence of a blocker of TRPV4 channels. In small human pulmonary arteries, ACh and sodium nitroprusside (SNP) induced concentration-dependent relaxations, blocked by endothelial cell removal, in the presence of an inhibitor of NO synthase and the KCa3.1 channel blocker TRAM-34. GSK1016790A induced relaxation of human pulmonary arteries with endothelium, but failed to relax arteries without endothelium. The findings suggest that TRPV4 channels are involved in endothelium-dependent relaxation and likely regulate pulmonary vascular tone by modulating NO release.

## Simple Summary

Lung diseases, such as pulmonary hypertension and acute lung injury, are driven by abnormal narrowing of blood vessels and damage to the vessel lining, which can lead to heart failure and impaired oxygen delivery. In this study, we investigated how a specific calcium pathway in blood vessel cells regulates vasorelaxation by releasing nitric oxide and activating potassium channels. Using mouse models and human lung blood vessels, we found that activation of this calcium pathway strongly promotes vessel relaxation by increasing nitric oxide production and electrical signaling that relaxes the smooth muscle cells. This mechanism remained functional even when certain potassium channels were absent, which indicates the presence of compensatory protective pathways. Our results show that this calcium signaling system plays a central role in controlling lung blood vessel tone under both healthy and disease conditions. These findings are valuable because they identify potential therapeutic targets that could be used to relax lung blood vessels in pulmonary hypertension.

## 1. Introduction

Acute respiratory distress syndrome (ARDS) is a severe and sometimes fatal form of respiratory failure that manifests as diffuse alveolar damage, inflammation, pulmonary edema, and severe hypoxemia not explained by cardiac failure [1]. ARDS develops from either viral (e.g., SARS-CoV-2) or non-viral infection (e.g., sepsis), can be associated with chronic disorders, and is often complicated by an increase in pulmonary vascular resistance and pulmonary hypertension (PH) that, in turn, leads to right ventricular dysfunction and right ventricular failure [2]. Vascular remodeling is similar in PH and ARDS. Thus, PH drugs (e.g., ambrisentan, sildenafil, and riociguat) have been tested for the treatment of ARDS [3].

ARDS and endothelium-dependent vascular relaxation in pulmonary arteries are linked by the disruption of normal vascular regulatory mechanisms in the lungs. Endothelial dysfunction in ARDS can lead to vasoconstriction, impaired NO production, and the development of PH, all of which contribute to the pathophysiology of ARDS and its impact on oxygenation and overall lung function. Understanding this link is crucial for the management and treatment of ARDS patients [4,5]. Endothelial-dependent vascular relaxation of pulmonary arteries is a crucial mechanism that helps maintain proper blood flow and oxygenation in the lungs. It involves the release of NO from endothelial cells (via endothelial nitric oxide synthase (eNOS)), which subsequently relaxes pulmonary smooth muscle cells, causing vasodilation and increased blood flow [5]. Within the context of endothelial-dependent vascular relaxation, the concerted interplay of caveolin-1 (a protein that regulates shear stress vascular relaxation [6]), eNOS, caveolae, transient receptor potential (TRP) channels, and calcium-activated potassium (K^+^) channels constitutes a multifaceted regulatory network in vascular relaxation [7].

KCa2.3 is a small-conductance Ca^2+^-activated K^+^ channel, and KCa3.1 (KCNN4) is an intermediate-conductance Ca^2+^-activated K^+^ channel. Both are calmodulin-gated and key effectors of endothelial hyperpolarization [8]. KCa3.1 has been implicated in vascular remodeling and atherogenesis (mouse and human data in the prior literature), and KCa3.1 expression/function in endothelial and smooth muscle compartments has been described—supporting translational interest in KCa3.1 as a therapeutic target [9]. TRPV4 is an upstream Ca^2+^ entry channel that couples to KCa channels to produce EDH-type vasodilation (endothelium-dependent hyperpolarization [10]). TRPV4 is expressed in the lung, specifically in vascular endothelial cells that regulate blood flow and vascular permeability; airway epithelial cells involved in mucus production, ciliary beating, and ion transport for lung fluid homeostasis; and alveolar epithelial cells responsible for gas exchange. TRPV4 inhibitors demonstrated vasculoprotective benefits by preventing vascular leakage and enhancing blood oxygenation [11].

The KCa2.3, KCa3.1, and TRPV4 channels in endothelial cells are likely involved in vascular relaxation. They achieve this by regulating membrane potential, calcium levels, and the release of vasodilatory molecules such as NO, thereby relaxing pulmonary blood vessels and improving blood flow in pulmonary circulation [12].

Activated by G-Protein-Coupled Receptor stimulation and Ca^2+^-signaling, the endothelial calcium/calmodulin-regulated intermediate-conductance K^+^ channel, KCa3.1, and the cation-permeable channels of the transient receptor potential (TRP) gene family mediate vascular relaxation. The activation of the endothelial Ca^2+^-permeable TRP (vanilloid) type 4 (TRPV4) receptors with selective openers, such as GSK1016790A, results in Ca^2+^ entry and vascular relaxation via Ca^2+^-dependent eNOS, induces acute lung injury, and can cause circulatory collapse [13].

TRAM-34 has previously been described as a selective KCa3.1 blocker that can be used to treat ARDS. Blockade of the KCa3.1 channel reduces lung injury in ARDS [1,13].

UCL1684 is a non-peptidic blocker of the apamin-sensitive Ca^2+^-activated K^+^ channel (KCa2.1 and KCa2.3) blocker [5].

Previously, functional studies (ex vivo human coronary arterioles and other human resistance vessels) have shown that TRPV4 activation mediates flow-induced dilation and that TRPV4 phosphorylation and regulation affect human arteriolar vasodilation; later human arteriolar work also implicates reactive oxygen species (ROS) generated by nicotinamide adenine dinucleotide phosphate (NADPH) oxidase in modulating TRPV4 signaling [14]. We have previously shown that a genetic deficiency of KCa3.1 channels reduces lung damage caused by pharmacological activation of calcium-permeable TRPV4 channels and that inhibition of KCa3.1 channels may have therapeutic potential in conditions characterized by abnormally high endothelial calcium signaling, barrier disruption, lung edema, and pulmonary circulatory collapse [13]. Recently, it has been shown that selective TRPV4 channel inhibitors prevent channel activation and reduce pulmonary edema in heart failure patients by modulating calcium influx [15].

In the present study, we hypothesized that the KCa2.3, KCa3.1, and TRPV4 channels in endothelial cells mediate vascular relaxation in mouse and human pulmonary arteries.

In the present study, we tested our hypotheses regarding the pharmacological effects of TRPV4 (GSK1016790A incubation), KCa3.1 (TRAM-34 incubation), and KCa2.3 (UCL1684 incubation) channels and pulmonary vascular relaxation by measuring arterial relaxation using wire myography. We believe that targeting molecules for TRPV4, KCa3.1, or KCa2.3 can be used to treat PH and ARDS. In addition, we aimed to achieve the therapeutic goal of vascular relaxation by modulating TRPV4, KCa3.1, and KCa2.3 channels in human pulmonary arteries.

In this study, we sought to examine the pharmacological effects of agents targeting TRPV4 (GSK1016790A), KCa3.1 (TRAM-34), and KCa2.3 (UCL1684) channels on pulmonary vascular relaxation, as assessed by wire myography. Our study suggests that compounds targeting TRPV4, KCa3.1, or KCa2.3 are promising candidates for therapeutic interventions to mitigate conditions such as PH and ARDS.

## 2. Materials and Methods

### 2.1. Data and Statistical Analysis

The data were summarized as mean ± SEM, with a significance level of *p* < 0.05, and n represents the number of individual animals or patients. The two-way analysis of variance (ANOVA) was used to compare the means of functional study observations where appropriate. Multiple-comparison analysis with Tukey’s post hoc tests were performed where appropriate. Statistical analysis of the data was performed with R-4.5.2 for Windows; *p* < 0.05 was considered statistically significant. “Smoking Status” was not used as a covariate in the ANOVA comparison because no patients had chronic obstructive pulmonary disease [16].

### 2.2. Animal Handling

*Kcnn4*^−/−^ mice were generated by targeted deletion of the Kcnn4 gene encoding the intermediate-conductance Ca^2+^-activated K^+^ channel (KCa3.1/IKCa), as previously described [13]. These mice are viable and fertile, but exhibit altered endothelial hyperpolarization and impaired endothelium-dependent relaxation in several vascular beds, including resistance arteries. Developmental compensation may occur, particularly via enhanced NO signaling or recruitment of alternative KCa channels, which must be considered when interpreting pharmacological data. Adult male *Mus musculus* BALB/c mice were humanely euthanized through decapitation followed by exsanguination, as previously described [13].

### 2.3. Tissue Preparation

Human pulmonary arteries, approximately 2 mm in length, were mounted on 40 µm steel wires in myographs (Danish Myotechnology, Aarhus, Denmark) for isometric tension recording. The vessels were equilibrated in oxygenated physiological salt solution (PSS) at 37 °C for 30 min and, by stretching, normalized to a lumen diameter (d100) equivalent to 100 mm Hg (23 mm Hg in human pulmonary arteries), after which the tension was set to 90% × d100, recording as previously described [17,18]. A total of 32 adults (24 men and 8 women; mean age, 68.4 ± 1.4 years) who had undergone surgery for lung carcinoma at the Department of Cardiac, Thoracic, and Vascular Surgery, Hospital of the Lithuanian University of Health Sciences, Kaunas, from March 2021 to July 2023, were included in the study. The research was carried out in accordance with the Declaration of Helsinki (2000) of the World Medical Association. The study protocol was approved by the Regional Biomedical Research Ethics Committee of the Lithuanian University of Health Sciences (Nos. BE-2-63, 1 August 2019, and P1-BE-2-39/2022, 9 May 2022), and each participant provided informed written consent. Among the study population, 25 individuals currently smoked and had a history of >10 pack-years, and 7 were never smokers. Small pulmonary arteries were carefully dissected under a microscope, the surrounding tissue removed and used for further experiments extemporaneously.

### 2.4. Wire Myography

We normalized the arterial segments and carefully followed the standard Mulvany–Halpern normalization protocol for pulmonary vessels. The vessels were constricted with a thromboxane mimetic that induces vasoconstriction by binding to the thromboxane A2 receptor U46619. In the series of experiments, the first relaxation after incubation with a drug was performed with ACh (10^−10^ M–10^−5^ M), followed by SNP (10^−10^ M–3 × 10^−5^ M)-induced relaxation, unless stated otherwise [18]. The endothelial cells were removed by introducing into the lumen a human scalp hair and rubbing back and forth several times, where appropriate, and the success of the denudation was tested with the ACh vascular relaxation test [19].

### 2.5. Experimental Protocol

#### 2.5.1. Incubation with TRAM-34 and UCL1684

U46619-contracted small pulmonary arteries were incubated with either TRAM-34, UCL1684, or both. The arteries were later relaxed with either ACH or SNP. We used TRAM-34 to block KCa3.1 channels and UCL1684 to block KCa2.3 channels. Calcium-activated K^+^ (KCa) channels open in response to increases in cytosolic calcium and modulate calcium-signaling and membrane potential in cells. The KCa2.3 and KCa3.1 channels initiate the endothelium-derived hyperpolarization (EDH) response in vascular endothelium, which causes relaxation of vascular smooth muscle cells via hyperpolarization, mediated by closure of voltage-gated calcium channels [8,20]. We conducted these experiments to investigate whether blocking both KCa3.1 and KCa2.3 can eliminate vascular relaxations that rely on vascular endothelial function. For wire myography experiments, U46619 was used as a constrictor, providing a stable contraction and allowing the construction of relaxation curves. U46619 was dissolved in ethanol, and, to avoid solvent interference with the NO microelectrodes, phenylephrine (PE) contraction was used to obtain simultaneous measurements of NO and relaxation, as described previously [21]. Briefly, an NO-sensitive microelectrode (ISONOP30, World Precision Instruments, Stevenage, UK) was calibrated in increasing concentrations of NO solution. Then, the electrode was introduced into the lumen of the pulmonary artery mounted in a single-channel wire myograph, allowing simultaneous registration of NO concentration and contractility [21,22].

#### 2.5.2. Incubation with GSK1016790A

We used GSK1016790A to activate TRPV4 channels in pulmonary endothelial cells [23]. Stretch-activated TRPV4 channels in endothelial cells elevate Ca^2+^ levels, triggering the production of NO by endothelial NO synthase (eNOS). This NO then permeates into the neighboring layer of smooth muscle cells (SMCs), initiating cyclic guanosine monophosphate (cGMP)-mediated signaling to activate myosin light chain phosphatase (MLCP). Consequently, this cascade reduces contractile force and promotes vasodilation [24]. We tested whether the KCa3.1 channel blockade with TRAM-34 can counteract the effects of TRPV4 channel activation with GSK1016790A, which induces vascular relaxation.

#### 2.5.3. TRPV4 Inhibition (HC067047)

HC067047 is a highly selective TRPV4 channel blocker commonly used to assess TRPV4-dependent Ca^2+^ signaling in vascular tissues. In pulmonary arteries, HC-067047 effectively inhibits the TRPV4-mediated Ca^2+^ influx, NO release, and endothelium-dependent relaxation induced by TRPV4 channel activators such as GSK1016790A. Concentrations in the low micromolar range are sufficient to block TRPV4 without significant off-target effects [25].

#### 2.5.4. NO Synthase Inhibition (L-NNA)

Nω-nitro-L-arginine (L-NNA) is a competitive NO synthase inhibitor used to assess the contribution of NO to vascular relaxation. In pulmonary arteries, L-NNA markedly reduces ACh and TRPV4-dependent relaxations, indicating a central role for eNOS-derived NO downstream of endothelial Ca^2+^ entry. L-NNA is routinely applied at 100–300 µM to ensure effective NOS inhibition in isolated vessel preparations [26].

#### 2.5.5. Non-Selective TRP Inhibition (Ruthenium Red)

Ruthenium red is a broad-spectrum TRP channel blocker that inhibits multiple Ca^2+^-permeable TRP channels, including TRPV4. It is commonly used to confirm TRP channel involvement in Ca^2+^-dependent vascular responses. In pulmonary arteries, ruthenium red suppresses endothelial Ca^2+^ influx and attenuates NO-dependent relaxation, supporting the involvement of TRP-mediated Ca^2+^ entry in endothelial signaling [27].

#### 2.5.6. Sarcoplasmic Reticulum Ca^2+^-ATPase Inhibition (Cyclopiazonic Acid, CPA)

Cyclopiazonic acid (CPA) inhibits sarcoplasmic/endoplasmic reticulum Ca^2+^-ATPase (SERCA), leading to depletion of intracellular Ca^2+^ stores and activation of store-operated and TRP-mediated Ca^2+^ entry. In pulmonary arteries, CPA induces endothelium-dependent relaxation through sustained endothelial Ca^2+^ elevation, activation of eNOS, and stimulation of KCa3.1-dependent hyperpolarization. The loss of CPA-induced relaxation following NOS inhibition or KCa3.1 blockade supports a mechanistic link between store depletion, Ca^2+^ entry, NO release, and KCa3.1 activation [28,29].

### 2.6. Substances Used in the Experiments

All chemicals were bought from Sigma-Aldrich (St. Louis, MO, USA). The drugs were prepared as stock solutions in distilled water (ACh, LNNA, and ruthenium red), ethanol (U46619), or DMSO (GSK1016790A, UCL1684, and cyclopiazonic acid). The stock solutions were further diluted in distilled water to ensure that the final organ bath concentration of the solvent’s ethanol and DMSO was below 0.01%.

### 2.7. Group Size and Selection Criteria

Each experiment was performed 5 times on vessels harvested from different patients (unless otherwise stated). Human pulmonary arteries were dissected from lung tissues from adults undergoing lung resection surgery for lung carcinoma after obtaining their signed informed consent.

## 3. Results

**Figure 1:**
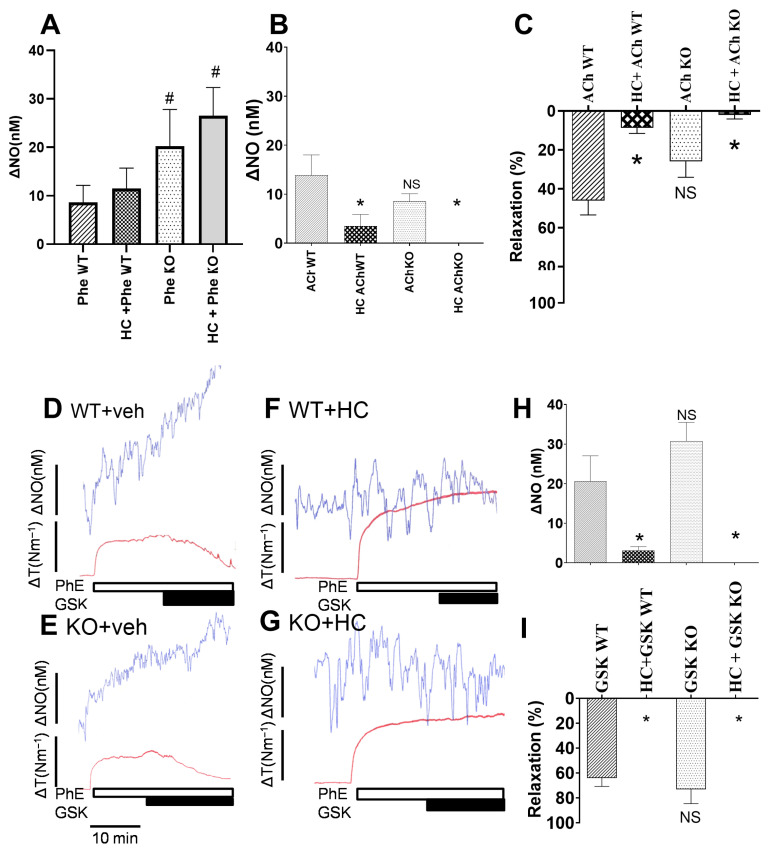
Simultaneous measurements of NO relaxation in pulmonary arteries from wild-type (wt) and knockout mice (KO) deficient in KCa3.1 channels. (**A**) Increase in NO induced by phenylephrine (PhE, 10^−7^ M) in the absence and the presence of the TRPV4 channel inhibitor, HC067047 (HC, 10^−6^ M). Acetylcholine (ACh, 10^−5^ M) induced a simultaneous increase (**B**) in NO and (**C**) relaxation in the absence and the presence of HC067047 (HC, 10^−6^ M). (**D**–**G**) Original recordings showing simultaneous measurements of NO (blue traces) and changes in contractile force measured as tension (ΔT, Nm^−1^). GSK101697A (10^−8^ M) increases NO and causes relaxation in pulmonary arteries from (**D**) wt and (**E**) *Kcnn4*^−/−^ KO mice in the presence of vehicle (Veh), but not in the presence of HC067047 (**F**,**G**). (**H**,**I**) Average increases in NO and relaxation induced by GSK101697A (10^−8^ M) in pulmonary arteries from wt and *Kcnn4*^−/−^ KO mice in the absence and the presence of HC067047 (HC, 10^−6^ M). The experiments were conducted in PSS. Results are means ± SEM of 4–7 mice. Two-way ANOVA: * *p* < 0.05 from vehicle control; # *p* < 0.05 comparing KO to wt mice; NS, non-significant comparing KO to wt mice.

In proximal pulmonary arteries from wt and *Kcnn4*^−/−^ knockout mice, we simultaneously measured NO and contractility. Phenylephrine-induced increases in NO concentration were unaltered in the presence of a blocker of TRPV4 channels, HC06704 (Figure 1A), but the NO response was increased in pulmonary arteries from *Kcnn4*^−/−^ KO compared to wt. In pulmonary arteries from *Kcnn4*^−/−^ KO mice, ACh-induced increases in NO and relaxation were slightly reduced (*p* = 0.10 and *p* = 0.06, respectively) compared with wt, and significantly inhibited in the presence of a blocker of TRPV4 channels, HC06704 (Figure 1B,C). Original traces showed that an activator of TRPV4 channels, GSK1016790A, increased NO and relaxation to the same degree in pulmonary arteries from wt and *Kcnn4*^−/−^ KO mice (Figure 1D,E), and these responses were abolished in the presence of a blocker of TRPV4 channels, HC06704 (Figure 1F,G). The average results showed no differences in the increase in NO and in relaxation in response to GSK1016790A between pulmonary arteries from wt and *Kcnn4*^−/−^ mice (Figure 1H,I), and these responses were significantly inhibited by the TRPV4 channel blocker, HC06704.

**Figure 2:**
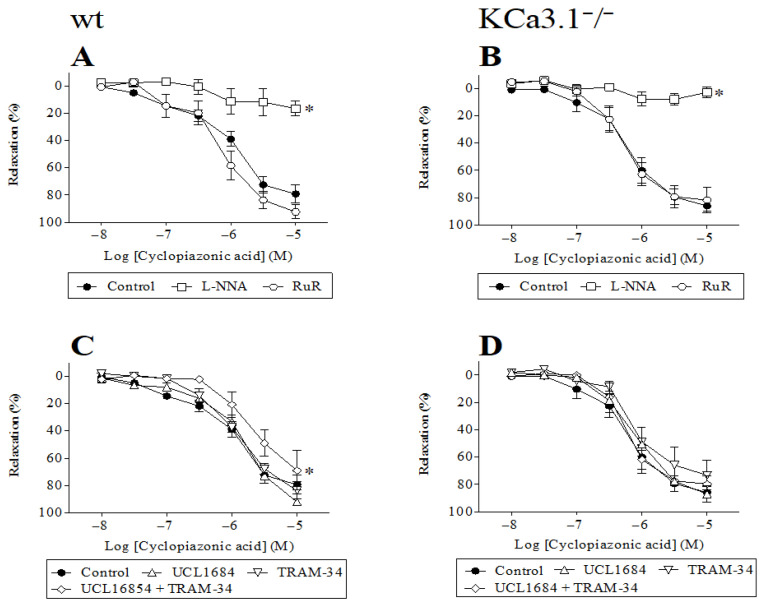
The effect of cyclopiazonic acid on mouse pulmonary arteries. Cyclopiazonic acid-induced relaxation in the presence and absence of L-NNA (300 µM), ruthenium red (RuR, 1 µM), UCL1684 (1 µM), and TRAM-34 (1 µM), inhibitor of eNOS, blocker of TRPV4 channels, and KCa2.3 and KCa3.1 channels, respectively, in wild-type (wt, (**A**,**C**)) and *Kcnn4*^−/−^ mice (**B**,**D**). Results are means ± SEM (*n* = 3–5). Two-way ANOVA: * *p* < 0.05 from vehicle control.

**Figure 3:**
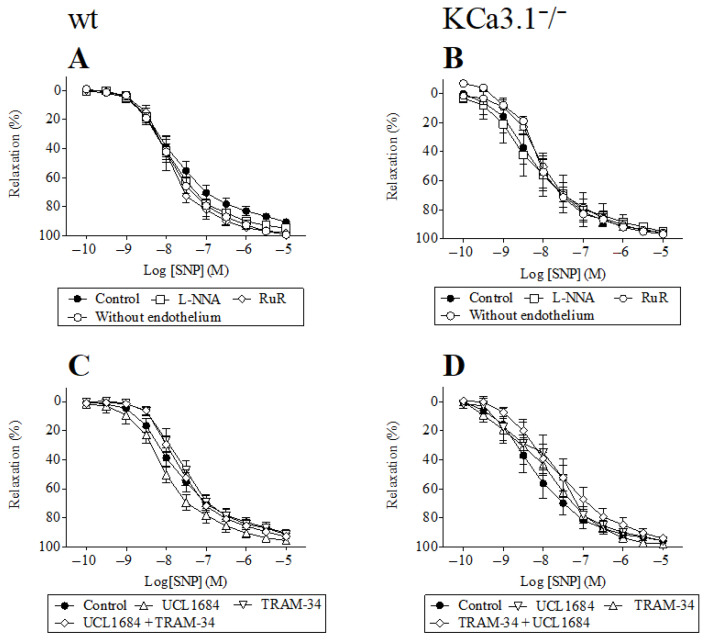
Effect of SNP on the mouse pulmonary artery. SNP induced relaxation in the presence and absence of L-NNA (300 µM), ruthenium red (RuR, 1 µM), UCL1684 (1 µM), and TRAM-34 (1 µM), inhibitors of eNOS, TRPV4 channels, and KCa2.3 and KCa3.1 channels, respectively, in wild-type (**A**,**C**) and *Kcnn4*^−/−^ KO mice (**B**,**D**). Results are means ± SEM (*n* = 6). Two-way ANOVA.

We also found that, in both wild-type (wt) and KCa3.1 knockout mice (*Kcnn4*^−/−^), cyclopiazonic acid-induced relaxation can be inhibited by L-NNA, whereas, in the presence of ruthenium red, UCL1684, and TRAM-34, cyclopiazonic acid and SNP cause dose-dependent vascular relaxation (Figure 2 and Figure 3).

**Figure 4:**
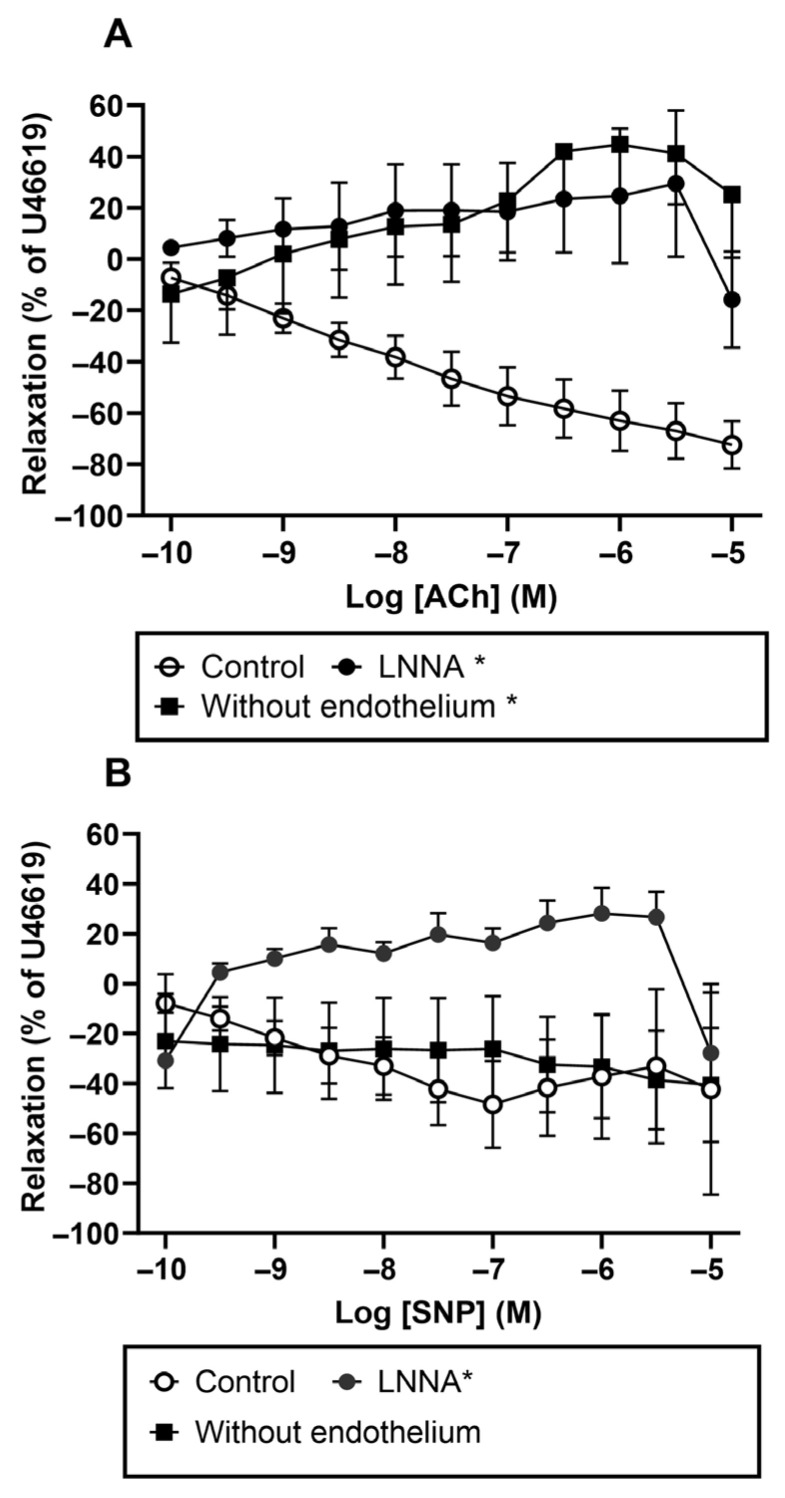
Effect of endothelial cell removal and inhibition of NOS on (**A**) acetylcholine (ACh) and (**B**) sodium nitroprusside (SNP) in human pulmonary arteries. (**A**) U46619-contracted small pulmonary arteries incubated with LNNA and relaxed with Ach, small pulmonary arteries with removed endothelium versus control U46619 contraction and Ach relaxation. Data are means ± SEM (*n* = 3–6); *p* < 0.05 between control and LNNA incubation and pulmonary arteries with removed endothelium groups, two-way ANOVA. (**B**) U46619-contracted small pulmonary arteries incubated with LNNA and relaxed with SNP, small pulmonary arteries with removed endothelium, and control U46619 contraction and SNP relaxation. Data are means ± SEM (*n* = 3–6); * *p* < 0.05 compared to the control arteries with endothelium and pulmonary arteries without endothelium, two-way ANOVA.

We also conducted experiments on human pulmonary arteries. We found that U46619-contracted small human pulmonary arteries relax in response to ACh only when the endothelium is intact; this relaxation was inhibited with N^G^-nitro-L-Arginine (LNNA). We obtained somewhat different results with SNP relaxation: endothelial removal and LNNA incubation did not inhibit pulmonary vascular relaxation, indicating that SNP elicits pulmonary vascular relaxation via an endothelium-independent mechanism (Figure 4).

**Figure 5:**
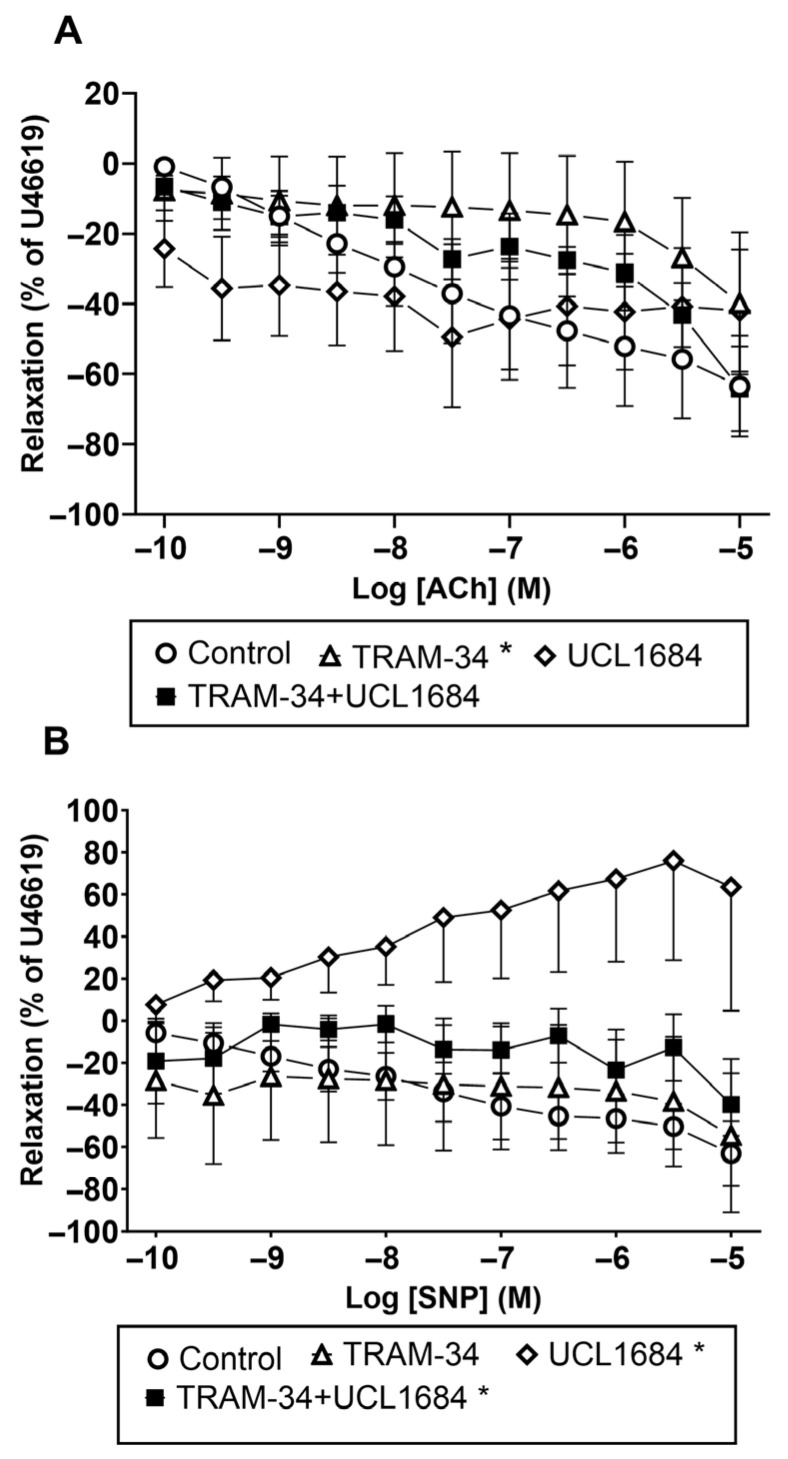
Effects of calcium-activated potassium channel blockers on acetylcholine (ACh) and sodium nitroprusside (SNP) relaxations in human pulmonary arteries. U46619-contracted small human pulmonary arteries relaxed with increasing concentrations of (**A**) ACh and (**B**) SNP in the absence (vehicle control) and in the presence of TRAM-34, UCL1684, and TRAM-34 plus UCL1684. Data are means ± SEM (*n* = 5–6). Two-way ANOVA: * *p* < 0.05 versus the control curve. Original traces are shown in Supplementary Figure S1.

Furthermore, we found that KCa3.1 blockade with TRAM-34 significantly inhibited Ach-induced vascular relaxation (Figure 5A). The KCa2.3 blocker UCL1684 did not alter Ach-induced vascular relaxation. Combined blockade of KCa2.3 and KCa3.1 channels with TRAM-34 and UCL1684 did not produce additional inhibition beyond that observed with TRAM-34 alone (Figure 5A). We repeated similar experiments with the NO donor SNP and found that KCa2.3 blockade inhibits SNP-induced vascular relaxation. The combined blockade of KCa2.3 and KCa3.1 channels with UCL1684 and TRAM-34 also significantly inhibited the concentration–response curves for SNP (Figure 5B).

**Figure 6:**
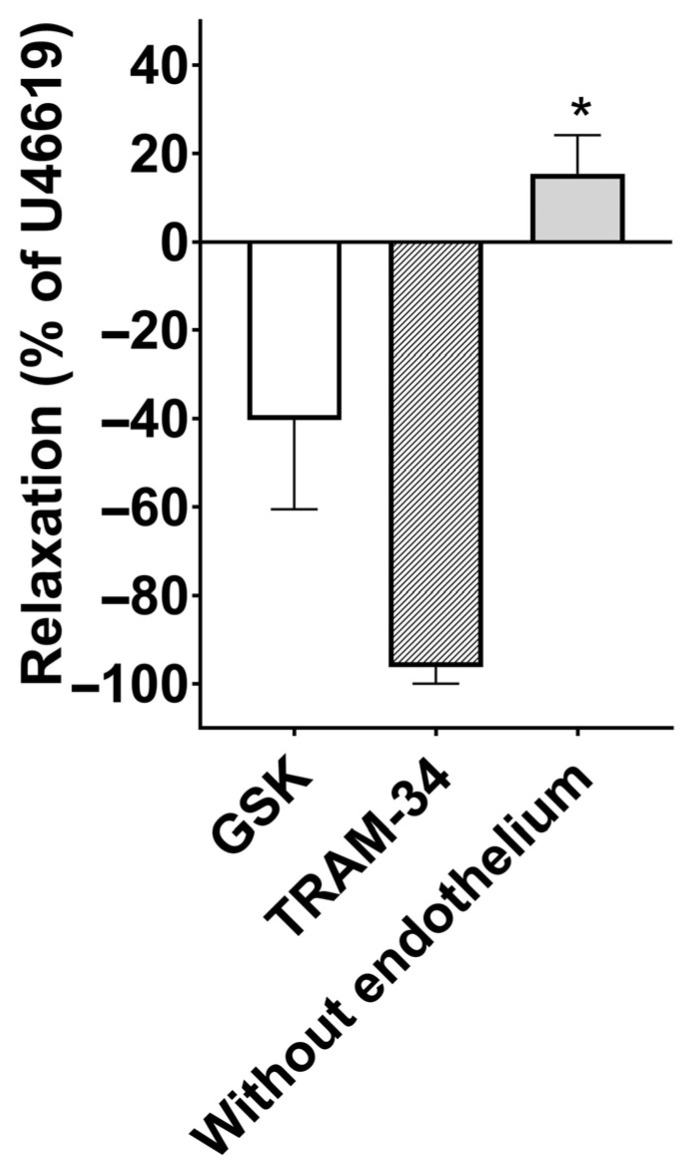
Effect of GSK1016790A (GSK, 10^−8^ M) on U46619-contracted human pulmonary arteries with and without endothelium, and with endothelium in the presence of the KCa3.1 channel blocker, TRAM-34. In preparations without endothelium, GSK induced contraction in U46619-contracted arterial segments. Data are means ± SEM (*n* = 4–5), * *p* < 0.05, one-way ANOVA.

Finally, we tested TRPV4 receptor activation in pulmonary endothelial cells using GSK1016790A. We found that U46619-contracted pulmonary arteries relax when the TRPV4 receptors are activated. This effect was converted to small contractions in arteries without endothelium, but not inhibited by KCa 3.1 blockade (TRAM-34 incubation) (Figure 6).

## 4. Discussion

We show that KCa3.1 blockade with TRAM-34 inhibits ACh-induced vascular relaxation in human pulmonary arteries, confirming a central role for endothelial KCa3.1 in endothelium-dependent vasodilation. Combined blockades of KCa2.3 and KCa3.1 produced a greater inhibitory effect, which can be explained by their distinct spatial organization within the endothelium. KCa2.3 channels are predominantly localized at endothelial cell junctions, whereas KCa3.1 channels are enriched in endothelial projections traversing the internal elastic lamina and positioned near myoendothelial gap junctions, enabling efficient electrical coupling to vascular smooth muscle cells [8,20].

In contrast to ACh, KCa2.3 blockade significantly attenuated relaxation to SNP, while KCa3.1 blockade caused a rightward shift of the SNP concentration–response curve. These effects differ from those observed with ACh and are consistent with the endothelium-independent mechanism of SNP, which acts directly on vascular smooth muscle [30]. The sensitivity of SNP-induced relaxation to KCa2.3 blockade suggests a contribution of KCa2.3 channels beyond classical endothelial signaling. The KCa channels of small conductance can be constitutively expressed in certain vascular beds, e.g., renal arteries [31], and can be induced in the smooth muscle layer in connection with arterial remodeling [32]. Indeed, the human pulmonary artery data should be interpreted in the context of the patient cohort’s advanced age (mean 68.4 years) and high smoking prevalence (78%). Both aging and chronic smoking are independently associated with endothelial dysfunction, reduced NO bioavailability, oxidative stress, and impaired Ca^2+^–dependent endothelial signaling, which could modify TRPV4–KCa pathway responses in ex vivo vessels [33,34]. Age-related endothelial dysfunction has been linked to impaired TRPV4-mediated dilation in other vascular beds, supporting the notion that TRPV4 signaling and NO release decline with age. Chronic smoking similarly induces persistent oxidative injury and endothelial dysregulation that may blunt NO-dependent and hyperpolarizing mechanisms. These factors may contribute to differential sensitivity to TRPV4 and KCa modulators in human pulmonary arteries compared with younger, naive animal models, highlighting that comorbidities likely influence vasorelaxant responses [33,35]. Together, these findings support recent proposals that KCa2.3- and KCa3.1-dependent mechanisms represent relevant therapeutic targets in cardiovascular disease [8,36].

Previous studies have demonstrated that KCa2.3 and KCa3.1 channels are expressed in the endothelium of human pulmonary arteries and mediate vasorelaxation, and these channels have been proposed as therapeutic targets in PH [4,37]. Our findings reinforce this concept and further demonstrate that pharmacological inhibition of KCa3.1 with TRAM-34 effectively attenuates endothelium-dependent relaxation. This is of particular interest because KCa3.1 has been implicated in hypoxia-induced pulmonary vascular remodeling through ERK/p38 MAP kinase signaling and has also been proposed as a therapeutic target in ARDS-like disease models [1,38].

TRPV4 has previously been shown to form a Ca^2+^ signaling complex with ryanodine receptors and large-conductance Ca^2+^-activated K^+^ (BKCa) channels in vascular smooth muscle, promoting hyperpolarization and vasodilation via Ca^2+^-induced Ca^2+^ release [39]. Based on this model, we investigated whether interference with downstream KCa signaling could counteract TRPV4-mediated pulmonary vascular relaxation.

Despite genetic deletion or pharmacological blockade of BKCa channels, vasorelaxation is often preserved, suggesting compensatory mechanisms within pulmonary circulation. These adaptations include increased reliance on endothelial NO signaling and enhanced contributions from alternative Ca^2+^-activated K^+^ channels, particularly KCa3.1 and KCa2.3, which help stabilize membrane potential and preserve vasodilatory capacity [12,40,41,42]. Such redundancy underscores the robustness of ion-channel-dependent regulation of pulmonary vascular tone.

Importantly, TRPV4 signaling does not uniformly engage all Ca^2+^-activated K^+^ channels in pulmonary arteries. Although TRPV4 is positioned upstream of multiple KCa channels, functional coupling preferentially favors endothelial intermediate-conductance KCa3.1 and small-conductance SKCa (including KCa2.3) channels, rather than smooth muscle BKCa channels. TRPV4-evoked Ca^2+^ microdomains within endothelial cells efficiently activate these channels, driving endothelium-dependent hyperpolarization and NO release, whereas activation of smooth muscle BKCa channels typically requires larger or distinct Ca^2+^ signals [43].

Consistent with this model, we found that KCa3.1 blockade did not inhibit TRPV4 agonist-induced pulmonary vascular relaxation, confirming that TRPV4-mediated relaxation is strictly endothelium-dependent, as removal of the endothelium abolished this response. Although TRPV4 inhibition has been proposed as a therapeutic strategy in respiratory diseases due to its role in pulmonary inflammation, vascular permeability, and lung edema [11], our data indicate that inhibition of KCa3.1 alone is insufficient to suppress TRPV4-mediated vasodilation.

A limitation of the present study is the limited direct comparability between the mouse and human vascular data. In the mouse experiments, vessels were precontracted with phenylephrine (PE), whereas human vessels were precontracted with U46619. This methodological difference may influence vascular responses and complicate the interpretation of interspecies comparisons. Furthermore, nitric oxide (NO)-related data were obtained only in mouse vessels, with no corresponding measurements in human tissues. Another limitation of this study is that the experimental data were obtained exclusively from healthy vessels. Although the discussion highlights the potential relevance of targeting TRPV4 in pathological conditions such as pulmonary hypertension (PH) and acute respiratory distress syndrome (ARDS), vascular tone regulation might be altered in these disease states. We, however, did not examine vessels from patients with PH or ARDS, nor did we investigate vessels from corresponding mouse models of these pathologies.

Taken together, our findings support a context-dependent therapeutic framework targeting the TRPV4–KCa signaling axis in pulmonary vascular disease. In PH, controlled activation of endothelial TRPV4 channels may enhance NO release and KCa3.1-mediated hyperpolarization, thereby promoting vasodilation and reducing pulmonary vascular resistance [44]. In contrast, in acute respiratory distress syndrome and pulmonary edema, excessive or dysregulated TRPV4 activation drives endothelial Ca^2+^ overload, barrier disruption, and pathological vascular leakage. In this setting, inhibition of TRPV4 or downstream KCa3.1 signaling may limit fluid extravasation, circulatory collapse, and lung injury [45].

## 5. Conclusions

In summary, our findings show that the KCa2.3, KCa3.1, and TRPV4 channels in endothelial cells mediate vascular relaxation in human pulmonary arteries. The KCa3.1 blockade can inhibit endothelium-dependent vascular relaxation, while both KCa2.3 and KCa3.1 mediate vascular relaxation in human pulmonary arteries. TRPV4 receptor-mediated vascular relaxation can be abolished with endothelial removal, but not with KCa3.1 blockade alone. The KCa2.3 and KCa3.1 channels are potential drug targets for the treatment of PH and ARDS.

## Data Availability

The original contributions presented in this study are included in the article/Supplementary Material. Further inquiries can be directed to the corresponding author.

## Supplementary Materials

The following supporting information can be downloaded at: https://www.mdpi.com/article/10.3390/biology15030292/s1, Figure S1: Effect of calcium-activated potassium channel blockers on Ach and SNP relaxations experimental trace data. (**A**)-U46619-contracted small pulmonary arteries incubated with TRAM-34, UCL1684, and TRAM-34, or UCL1684 alone with Ach relaxations. Experimental trace data. (**B**)-U46619-contracted small pulmonary arteries incubated with TRAM-34, UCL1684, and TRAM-34, or UCL1684 alone with SNP relaxations, control—U46619 contraction.

## Abbreviations

The following abbreviations are used in this manuscript:

ACh	Acetylcholine
ARDS	Acute respiratory distress syndrome
cGMP	Cyclic guanosine monophosphate
CPA	Cyclopiazonic acid
DMSO	Dimethyl sulfoxide
EDH	Endothelium-dependent hyperpolarization
eNOS	Endothelial nitric oxide synthase
L-NNA	Nω-Nitro-L-arginine
MLCP	Myosin light chain phosphatase
NADPH	Nicotinamide adenine dinucleotide phosphate, reduced
NO	Nitric oxide
PE	Phenylephrine
PH	Pulmonary hypertension
PSS	Physiological salt solution
ROS	Reactive oxygen species
SERCA	sarcoplasmic/endoplasmic reticulum Ca^2+^-ATPase
SMCs	Smooth muscle cells
SNP	Sodium nitroprusside
TRP	Transient receptor potential
TRPV4	Transient Receptor Potential Vanilloid 4
wt	Wild-type mice
